# The Diagnosis of Fevers

**Published:** 1851-10

**Authors:** 


*The Diagnosis of Fevers.*—If we had been asked some twenty years ago, to find an appropriate simile for the disease called Fever, we could have made no better comparison than with our serviceable friend Proteus. Fever, we should have said, is something of everything, nothing of its own: you never find it in the same costume. It transforms itself like a Harlequin, and when you think you have at last got it into a corner, it disappears into the depths below, and rises at the other end of the stage, in the likeness of Pantaloon. In Paris, in London, and in Edinburgh, it presents itself with a different physiognomy, or else, "like Cerberus, it is three gentlemen at once." Attempt to draw its portrait, and you make as great a medley as Blumenbach would have done, had he compounded an ideal skull out of his immense collection. It is as difficult as Etruscan, and is to be deciphered by guess-work, like the arrow-headed writing. It is like the sphynx, for it devours its interrogators, and gives them no clue to its riddle. And it is even worse than the sphynx, for that did yield to an Œdipus; whilst the solution of *this* enigma is yet to be found.

In some such despairing strain we should, twenty years ago, have spoken of a disease, which was said to be located in every organ of the body, and yet could be found no where; which presented all symptoms, yet apparently none twice over; which was cured in all ways, but seldom in the same way by any two persons; and which was designated by all sorts of names, the recommendation of each of which was, that it was as applicable as any other.

This uncertainty and discrepancy of observation does not, however, now prevail to anything like the extent it formerly did. The difficulties in the study of fevers have yielded, or appear to be yielding, before the close and incessant observation with which they have been regarded. The inquiries even of twenty years since, appear coarse and narrow by the side of our greater knowledge; and we look forward to the speedy coming of the time, when even our observations will seem superficial and ill-directed to the clearer light of advanced intelligence.—*Medico-Chirurgical Review.*

